# A pilot study exploring the association of morphological changes with 5-HTTLPR polymorphism in OCD patients

**DOI:** 10.1186/s12991-017-0126-6

**Published:** 2017-01-17

**Authors:** Shinichi Honda, Tomohiro Nakao, Hiroshi Mitsuyasu, Kayo Okada, Leo Gotoh, Mayumi Tomita, Hirokuni Sanematsu, Keitaro Murayama, Keisuke Ikari, Masumi Kuwano, Takashi Yoshiura, Hiroaki Kawasaki, Shigenobu Kanba

**Affiliations:** 1 Department of Neuropsychiatry, Graduate School of Medical Sciences, Kyushu University, 3-1-1 Maidashi Higashi-ku, Fukuoka, Japan; 2 Department of Mental Retardation and Birth Defect Research, National Institute of Neuroscience, National Center of Neurology and Psychiatry, Tokyo, Japan; 3 Kurume University Graduate School of Psychology, Fukuoka, Japan; 4 Department of Radiology, Graduate School of Medical and Dental Sciences, Kagoshima University, Kagoshima, Japan; 5 Department of Psychiatry, Faculty of Medicine, Fukuoka University, Fukuoka, Japan

**Keywords:** Hippocampus, Precentral gyrus, Frontal pole, Imaging genetics, Obsessive-compulsive disorder (OCD), Serotonin transporter gene, 5-HTTLPR, Voxel-based morphometry (VBM)

## Abstract

**Background:**

Clinical and pharmacological studies of obsessive-compulsive disorder (OCD) have suggested that the serotonergic systems are involved in the pathogenesis, while structural imaging studies have found some neuroanatomical abnormalities in OCD patients. In the etiopathogenesis of OCD, few studies have performed concurrent assessment of genetic and neuroanatomical variables.

**Methods:**

We carried out a two-way ANOVA between a variable number of tandem repeat polymorphisms (5-HTTLPR) in the serotonin transporter gene and gray matter (GM) volumes in 40 OCD patients and 40 healthy controls (HCs).

**Results:**

We found that relative to the HCs, the OCD patients showed significant decreased GM volume in the right hippocampus, and increased GM volume in the left precentral gyrus. 5-HTTLPR polymorphism in OCD patients had a statistical tendency of stronger effects on the right frontal pole than those in HCs.

**Conclusions:**

Our results showed that the neuroanatomical changes of specific GM regions could be endophenotypes of 5-HTTLPR polymorphism in OCD.

## Background

Obsessive-compulsive disorder (OCD) was made a disease independent of anxiety disorder in DSM-5. One of the reasons for this separation is that the biological bases of OCD and anxiety disorder are different [1].

Structural imaging studies have found neuroanatomical abnormalities in the cortico–striatal–thalamo–cortical (CSTC) circuits in OCD patients [2]. A recent voxel-based morphometry (VBM) systematic review suggested that widespread structural abnormalities may contribute to neurobiological vulnerability to OCD [3]. We previously found the presence of regional gray matter (GM) and white matter (WM) volume abnormalities in OCD patients [4].

Furthermore, positron emission tomography (PET) and functional magnetic resonance imaging (fMRI) have revealed abnormal activities in different nodes of the CSTC circuits in OCD patients compared with healthy controls (HCs) [2, 5]. In our previous fMRI study, we found decreased activations in several brain regions including the orbitofrontal cortex (OFC) [6] and a specific relationship between fMRI activation and symptom subtypes [7].

Meanwhile, family and twin studies have provided evidence for the involvement of a genetic factor in OCD. However, many linkage, association, and genome-wide association studies have failed to identify responsible genes [8, 9].

Molecular genetic studies have focused on some structures, including receptor and transporter proteins, in the serotonergic and dopaminergic system.

Based on transporter imaging findings, a PET study [10] found a decrease of serotonin transporter binding in the insular cortex in OCD patients. They suggested that dysfunction of the serotonergic system in the limbic area might be involved in the pathophysiology of OCD.

It is possible to hypothesize that a polymorphism in the transcriptional control region upstream of the 5-hydroxytryptamine (serotonin) transporter (5-HTT) coding sequence could be an important factor in conferring susceptibility to OCD [8, 11, 12]. The 5-HTTLPR consists of a 44-bp deletion/insertion yielding a 14-repeat allele (short; S) and a 16-repeat allele (long; L). The S allele reduces the transcriptional efficiency of the 5-HTT gene promotor, resulting in decreased 5-HTT expression and availability. Bloch et al. [11]. suggested the possibility that the L allele is associated with specific OCD subgroups such as childhood-onset OCD. In contrast, Lin et al. [12] found that OCD was associated with the SS homozygous genotype. Some researchers suggested that this L allele could be subdivided further to L_A_ and L_G_ alleles [13]. The L_G_ allele, which is the L allele with an *A*→*G* substitution (rs25531), is thought to be similar to the S allele in terms of reuptake efficiency [14]. Rocha et al. [15] found that the L_A_ allele was associated to OCD. Hu et al. [14] found that the L_A_L_A_ genotype was approximately twice as common in 169 whites with OCD than in 253 ethnically matched controls, and the L_A_ allele was twofold overtransmitted to the patients with OCD.

Despite the genetic and neuroanatomical importance, few studies of the etiopathogenesis of OCD have concurrently assessed genetic and neuroanatomical variables. We hypothesized that the widespread structural brain changes in OCD indicate the endophenotype of the 5-HTTLPR polymorphism. Therefore, the aim of this study was to investigate the association of genetic variations of the 5-HTTLPR with neuroanatomical changes in OCD.

## Methods

### Subjects

We studied 40 OCD patients (20 females and 20 males) who met DSM-IV [16] criteria for OCD and had no DSM-IV Axis I disorders except OCD and major depressive disorder as screened by the Structured Clinical Interview for DSM-IV (SCID). Patients who displayed a comorbid axis I diagnosis, neurological disorder, head injury, serious medical condition, or history of drug/alcohol addiction were excluded. We determined psychiatric diagnoses by a consensus of at least two psychiatrists after screening by SCID. Patients were recruited from among outpatients and inpatients of the Department of Neuropsychiatry, Kyushu University Hospital, Japan. Severity of OCD symptoms was assessed with the Yale-Brown Obsessive Compulsive Scale (Y-BOCS) [17]. Patients were also screened for the presence of depressive symptoms through the administration of the 17-item Hamilton Depression Rating Scale (HDRS) [18]. Forty HCs (26 females and 14 males) who were matched to the patients in age and sex were recruited from the staff of Kyushu University Hospital and related agencies. They had no DSM-IV Axis I disorders as determined by the SCID. They also had no current medical problems, psychiatric histories, neurological disorders, or mental retardation. Handedness was determined according to the Edinburgh Handedness Inventory for both OCD patients and HCs [19].

The study was approved by the local ethics committee <22-111, 491-01>, and each participating patient provided written informed consent after receiving a complete description of the study, which was approved by the institutional review board.

### MRI procedures

All imaging examinations were performed on a 3.0-T MRI scanner (Achieva TX, Philips Healthcare, Best, The Netherlands) with a standard head coil at the Department of Radiology, Kyushu University. T1-weighted images were acquired with a 3D T1-weighted turbo field echo sequence with the following parameters: repetition time (TR) = 8.2 ms, echo time (TE) = 3.8 ms, flip angle = 8°, matrix = 240 × 240, T1 inversion time = 1026 ms, field of view (FOV) = 240 × 240 mm, NSA = 1, slice thickness = 1 mm, number of slices = 190, and scan time = 320 s.

### VBM data processing

Acquired images were first converted from DICOM to NifTI-1 format using dcm2nii software (http://www.mccauslandcenter.sc.edu/mricro/mricron/dcm2nii.html). Data processing and examinations were performed with SPM8 software (developed under the auspices of the Functional Imaging Laboratory, The Wellcome Trust Centre for Neuroimaging at the Institute of Neurology at University College London, UK, http://www.fil.ion.ucl.ac.uk/spm/) in the environment of MATLAB (2011b ver., http://www.mathworks.co.jp/products/matlab/). AC–PC orientation was conducted on all T1-weighted data by an automatic process. Then, we applied the VBM8 toolbox (http://dbm.neuro.uni-jena.de/467/) for preprocessing the structural images by the VBM procedure. This VBM8 algorithm involves image bias correction, tissue classification, and normalization to the standard Montreal Neurological Institute (MNI) space using linear (12-parameter affine) and non-linear transformations. High-dimension DARTEL normalization, which is rather unbiased in its segmentation process, was used as anatomical registration with the default template provided in the VBM8 toolbox. Gray matter (GM) and white matter (WM) segments were modulated only by non-linear components, which allowed comparing the absolute amount of tissue corrected for individual brain volume, that is, correction for total brain volume.

Finally, modulated images were smoothed with a Gaussian kernel of 8 mm full width at half maximum. Although we used the East Asian Brains template in the process of affine regularization instead of European Brains, the default parameters were used in all other steps. Finally, 40 OCD patients and 40 HCs were assessed by structural MRI examinations with a 3.0-T MRI scanner.

### Genotyping

A 10-ml venous blood sample was collected in EDTA vacuum tubes. Samples were immediately frozen at −80 °C until extraction of genomic DNA from nucleated white blood cells. Genomic DNA was extracted from peripheral blood leukocytes using a Promega DNA Purification Kit (Promega, Madison, WI, USA).

The polymerase chain reaction (PCR) was used to amplify 5-HTTLPR polymorphism. Forward (5′-GGCGTTGCCGCTCTGAATGC-3′) and reverse (5′-GAGGGACTGAGCTGGACAACCAC-3′) primers were used to amplify a fragment including 5-HTTLPR [20]. These primers amplify a 529-bp fragment for the S allele and a 575-bp fragment for the L allele.

PCR amplification was carried out in a final volume of 15 μl consisting of 50–100 ng genomic DNA, 2.5 mM deoxyribonucleotides, 0.2 μM of forward and reverse primers, PCR buffer (2× GC Buffer I, Takara Bio Inc., Shiga, Japan), and 1.25 U of DNA polymerase (TaKaRa LA Taq, Takara Bio Inc.). Denaturation was carried out at 94 °C for 30 s, annealing at 64 °C for 30 s, and extension at 72 °C for 3 min for 40 cycles.

To identify L_A_ and L_G_ alleles, a two-step protocol was performed. Step I: determination of the L or S allele, as described above; and step II: digestion of this amplicon with *Hap*II (Takara Bio Inc.) restriction endonuclease. The assay was designed to include an invariant *Hap*II digest site located 94 bp from the end of the amplicon to provide an internal control for digestion/partial digestion. Products were separated on a 4.0% agarose gel (Agarose-LE, Classic Type, Nacalai Tesque, Inc., Kyoto, Japan) supplemented with ethidium bromide (0.01%, Nacalai Tesque) and visualized under ultraviolet light. After separation of the digestion products by electrophoresis, the following restriction fragment allele sizes were obtained: L_A_ (341, 126, 62 bp) and L_G_ (174, 167, 126, 62 bp).

### Statistical analysis

We conducted a two-sample *t* test, Chi square test, and Fisher’s exact test to test for differences in demographic variables between OCD patients and HCs as well as between different variants of the alleles of 5-HTTLPR in OCD patients.

The genotype frequencies of OCD patients and HCs were compared using Chi square test after checking the Hardy–Weinberg equilibrium.

We divided the patients into L_A_ allele carriers (SL_A_, L_A_L_G_, and L_A_L_A_) and non-L_A_ allele carriers (SS, SL_G_, and L_G_L_G_). Hu et al. [14] noted that the normalized (to SS genotype) expression value of the L_A_ allele was approximately double the values of the S and L_G_ alleles. Thus, we thought that expressions of genotypes including the L_A_ allele were higher than those of other genotypes.

Statistical analysis was performed with SPM8, which implemented a general linear model. First, we performed a two-sample *t* test to detect the difference in GM volume between patients with OCD and HCs. The initial voxel threshold was set to *P* < 0.001 uncorrected. Clusters were considered significant that fell below a cluster-corrected family-wise error (FWE), *P* = 0.05. Next, we performed a two-way factorial analysis of variance between the 5-HTTLPR polymorphism and GM volumes in the OCD patients and HCs. A two-way ANOVA test was applied to assess the relationship between 5-HTTLPR polymorphism (L_A_ or non-L_A_ allele carriers) and GM brain volume changes in the OCD patients and HCs. If a statistical difference was present, a post hoc *t* test was performed to detect the inter-group difference of brain regions. Age and sex were set as covariates in the statistical analysis. We used a threshold of *P* < 0.05 cluster-corrected family-wise error (FWE) and *P* < 0.001 uncorrected with expected voxels per clusters. The *P* < 0.001 value is commonly used in VBM-based OCD studies [21–23].

## Results

In demographic variables of age, gender, and handedness, OCD patients and HCs did not show any significant differences (Table 1). These variables also showed no significant difference between the genotypes of L_A_ allele carriers or non-L_A_ allele carriers in OCD patients (Table 2). OCD patients had significantly fewer years of education than HCs (Table 1). Non-L_A_ allele carriers, furthermore, had significantly fewer years of education than those of L_A_ allele carriers (Table 2). No significant differences were shown between the two genotypes in OCD regarding illness duration, age of onset, total Y-BOCS, or the 17-item HDRS (Table 2).

**Table 1:** Clinical and demographic characteristics of OCD patients and HCs

	OCD patients (*n* = 40)	HCs (*n* = 40)	*P* value
Age (years, mean ± SD)^a^	35.40 ± 12.07	39.70 ± 12.97	0.129
Gender (female/male)^b^	20/20	26/14	0.175
Handedness (right/left)^b^	37/3	39/1	0.305
Education (years, mean ± SD)^a^	13.69 ± 2.43	15.15 ± 1.35	0.001
Illness duration (years, mean ± SD)	11.33 ± 10.10		
Age of onset (years, mean ± SD)	23.98 ± 11.24		
Total Y-BOCS (total score, mean ± SD)	21.95 ± 6.32		
HDRS (17 items)^a^	6.08 ± 6.87	0.55 ± 0.88	0.000
5-HTTLPR^a^			
14/14	26	17	0.040
14/16	13	22	
16/16	1	1	
L_A_ allele carriers (SL_A_, L_A_L_G_, L_A_L_A_)^b^	10	20	0.021
Non-L_A_ allele carriers (SS, SL_G_, L_G_L_G_)^b^	30	20	

We found a significant difference between the OCD patients and HCs in the distribution of L_A_ allele carriers or non-L_A_ allele carriers of 5-HTTLPR polymorphism

^a^ *T* test

^b^Chi square test

**Table 2:** Clinical and demographic characteristics of non-L_A_ allele carriers and L_A_ allele carriers in OCD patients

	Non-L_A_ allele carriers (*n* = 30) (SS, SL_G_, L_G_L_G_)	L_A_ allele carriers (*n* = 10) (SL_A_, L_A_L_G_, L_A_L_A_)	*P* value
Age (years, mean ± SD)^a^	36.77 ± 12.50	31.30 ± 10.15	0.219
Gender (female/male)^b^	15/15	5/5	1.000
Handedness (right/left)^b^	27/3	10/0	0.411
Education (years, mean ± SD)^a^	13.23 ± 2.46	15.00 ± 1.70	0.042
Illness duration (years, mean ± SD)^a^	12.27 ± 10.75	8.50 ± 7.61	0.313
Age of onset (years, mean ± SD)^a^	24.53 ± 11.90	22.30 ± 9.30	0.593
Total Y-BOCS (total score, mean ± SD)^a^	22.75 ± 6.53	19.44 ± 5.15	0.176
HDRS (17 items)^a^	6.76 ± 7.61	4.10 ± 3.70	0.157

^a^ *T* test

^b^Chi square test

The genotype frequencies of our samples did not deviate significantly from the values predicted by the Hardy–Weinberg equation.

As for the genotypic distribution, 1/40 OCD patients (2.5%) and 1/40 HCs (2.5%) were LL homozygotes, 13/40 OCD patients (32.5%) and 22/40 HCs (55.0%) were LS heterozygotes, 26/40 OCD patients (65%) and 17/40 HCs (42.5%) were SS homozygotes, and 10/40 OCD patients (25.0%) and 20/40 HCs (50.0%) were L_A_ allele carriers. We found a significant difference between the OCD patients and HCs in the distribution of L_A_ allele carriers or non-L_A_ allele carriers of 5-HTTLPR polymorphism (*χ* ^2^=5.333, 1 *df*, *P* = 0.021; Table 1).

**Fig. 1:**
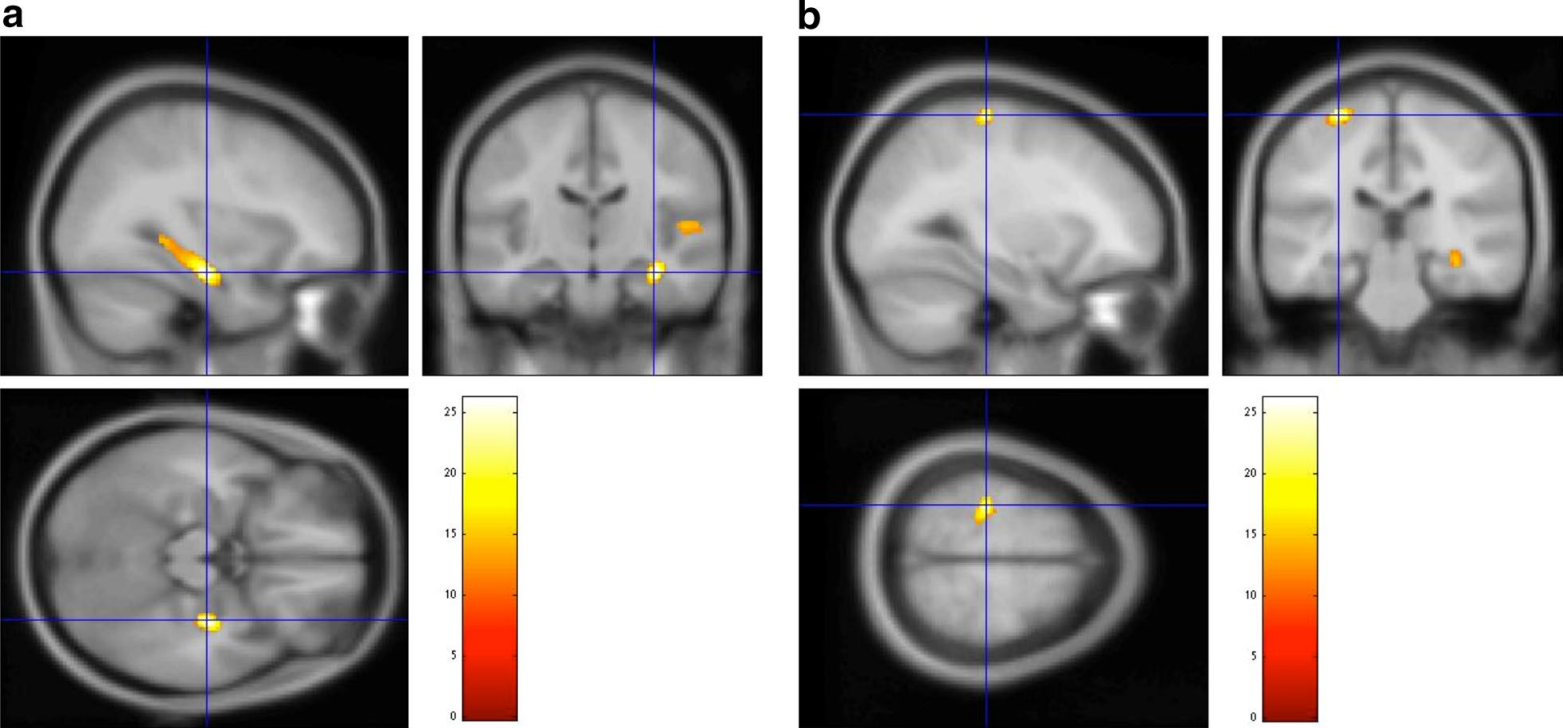
**a** OCD patients showed decreased GM volume in the right hippocampus compared to HCs. **b** OCD patients showed increased GM volume in the left precentral gyrus compared to HCs [*P* < 0.005, cluster-corrected family-wise error (FWE), 〈*k*〉 = 77.666]

In morphological changes in OCD, compared to the HCs, the OCD patients showed significant decreased GM volumes in the right hippocampus (extent threshold; *k* = 763 voxels, *P* < 0.05, FWE; Table 3; Fig. 1a) and increased GM volume in the left precentral gyrus (extent threshold; *k* = 797 voxels, *P* < 0.05, FWE; Table 3; Fig. 1b). In morphological changes associated with the 5-HTTLPR polymorphism, compared to L_A_ allele carriers, non-L_A_ allele carriers showed no significant GM volume difference.

**Table 3:** VBM analysis including association of variance between 5-HTTLPR polymorphisms and GM volumes in OCD patients and HCs

Regions	Brodmann area	Cluster size	*Z*	Talairach coordinates
				*x*, *y*, *z* (mm^2^)
Main effects				
Diagnosis effects (*P* < 0.05, FWE, 〈*k*〉 = 77.666)				
R hippocampus (OCD patients < HCs)		763	5.08	33, −16, −18
L precentral gyrus (OCD patients > HCs)	4	797	4.88	−28, −27, 64
Genotype effects				
No suprathreshold clusters				
Genotype-diagnosis interaction effects (*P* < 0.001, uncorrected, 〈*k*〉 = 63.146)				
R frontal pole	10	112	4.35	26, 50, −6

*R* right, *L* left, *FWE* family-wise error, 〈*k*〉 expected voxels per clusters

**Fig. 2:**
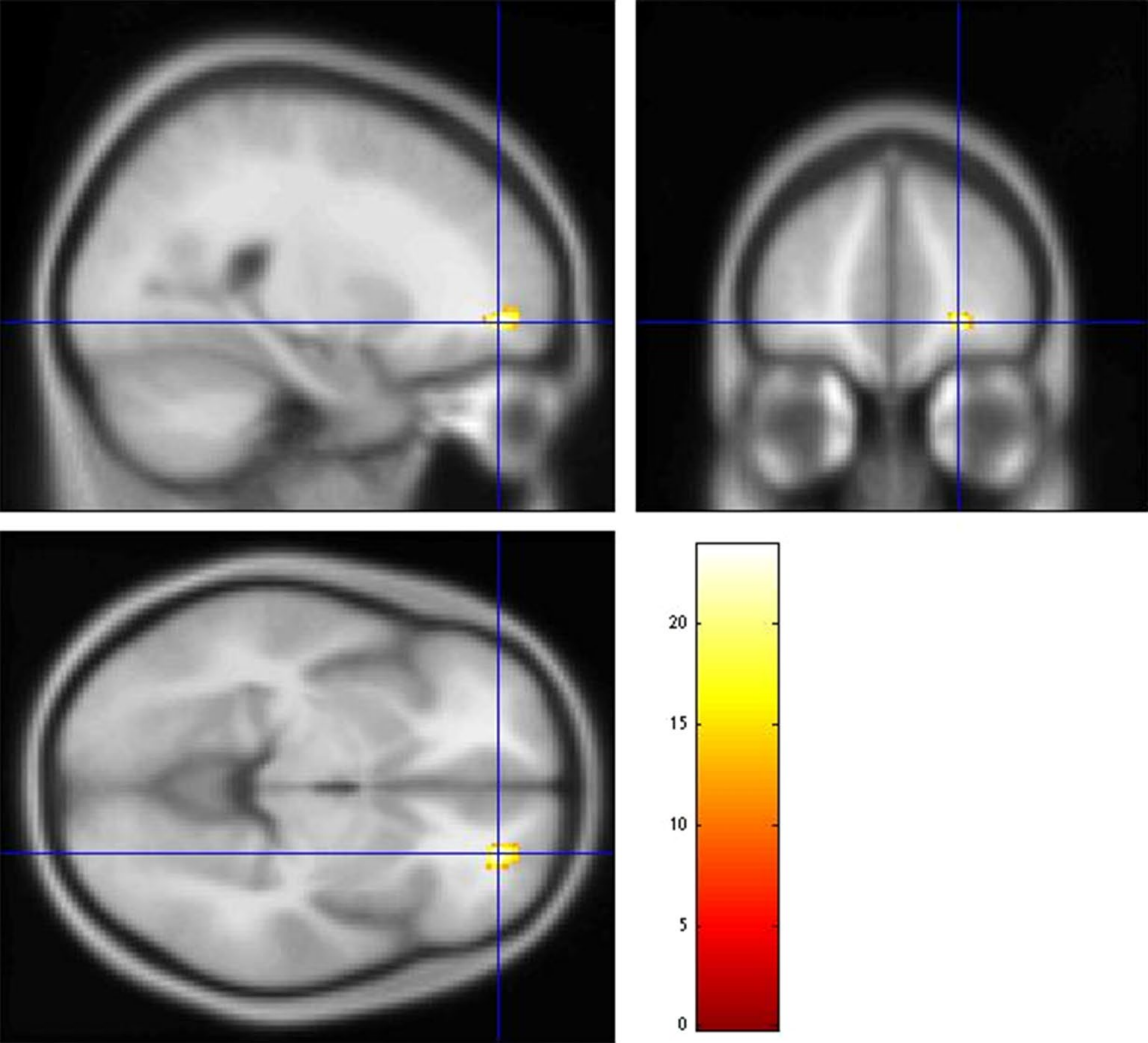
Results of genotype–diagnosis interaction effects on brain morphology. The stronger effects of 5-HTTLPR polymorphism on brain morphology in OCD patients than those in HCs were noted in the right frontal pole (*P* < 0.001, uncorrected, 〈*k*〉 = 63.146)

As of genotype–diagnosis interaction, although no voxels survived multiple comparison, we observed a tendency that 5-HTTLPR polymorphism in OCD patients had stronger effects on the right frontal pole than those in HCs (*P* < 0.001, uncorrected; Table 3; Fig. 2). The OCD patients with the L_A_ allele carriers of 5-HTTLPR polymorphism exhibited a statistical tendency of reduction of GM volumes in the right frontal pole compared to the HCs with the L_A_ allele carriers.

## Discussion

In the present study, we found that the OCD patients showed significant decreased GM volume in the right hippocampus and increased GM volume in the left precentral gyrus. Moreover, our study suggested that L_A_ allele carriers of the 5-HTTLPR polymorphism in OCD patients are associated with decreased GM volume in the right frontal pole.

Functional neuroimaging studies have been suggested that hippocampus might have an important role in the pathophysiology of OCD [24, 25]. On the other hand, structural imaging studies have been suggested that hippocampal alteration may play an important role in the pathophysiology of OCD [26, 27].

The precentral gyrus is a prominent structure on the surface of the posterior frontal lobe. It is the site of the primary motor cortex (Brodmann area 4). Several researchers have suggested that the precentral gyrus may be involved in the pathophysiology of OCD [28, 29]. Russo et al. [30] suggested that OCD might be considered as a sensory motor disorder where a dysfunction of sensory–motor integration might play an important role in the release of motor compulsions. Our results also showed that the precentral gyrus might be involved in the pathophysiology of OCD.

The frontal pole comprises the most anterior part of the frontal lobe that approximately covers BA10. During human evolution, the functions in this area resulted in its expansion relative to the rest of the brain [31]. Specifically, the functions include multi-tasking [32], cognitive branching [33], prospective memory [34], conflict resolution [35], and selection of sub-goals [36]. It is suggested that such a highly advanced cognitive function is affected in OCD [37, 38].

In the field of imaging genetics, many researchers reported [39–42] an association between the serotonin transporter gene and brain structure. Regarding OCD, Atmaca et al. [43] found a significant genotype-by-side interaction for the OFC.

In contrast to the previous result reported by Atmaca et al. [43], our result suggested that a liability in development of the central nervous system might have occurred in OCD patients who are L_A_ allele carriers. Frodl et al. [44] suggested that the high-activity L_A_ allele with its increased number of 5-HTT transporter proteins, concomitant decrease in serotonin levels, and reduced effects on neuroplastic processes might cause structural changes during major depression. With similar mechanism, the volume decrease in the right frontal pole might have occurred in OCD patients who are L_A_ allele carriers.

There are some limitations to this study. First, we divided the patients into L_A_ allele carriers (SL_A_, L_A_L_G_, and L_A_L_A_) and non-L_A_ allele carriers (SS, SL_G_, and L_G_L_G_). In the view of expression activity, it might be better to divide samples into L_A_L_A_ and others. Although we could not employ this division because our study included few L_A_L_A_ genotypes, the difference between L_A_L_A_ and other genotypes should be explored with larger samples in the future. In addition, our sample size was too small to identify the difference between the effects of L_A_ and non-L_A_ alleles on the brains of OCD patients and HCs. Thus, these findings should be considered preliminary until replicated in a larger sample.

The OCD patients had significantly fewer years of education than HCs, and non-L_A_ allele carriers had significantly fewer than L_A_ allele carriers. Education years might affect the difference in GM volumes if education years were proportional to high intelligence. In this study, we did not measure the intelligence quotient (IQ). Larger gray matter volumes are associated with higher IQ [45]. Ideally, the IQ should be measured and set as a covariate in the statistical analysis.

Although we examined 5-HTTLPR polymorphism as the sole candidate gene in this study, many other polymorphisms such as glutamate system genes and dopamine system genes [46, 47] may affect the brain morphology of OCD patients. We hope to explore an association between more candidate genetic polymorphisms and brain morphology in the future.

Moreover, the OCD patients were concurrently on medication. Our study was not designed to investigate medication effects. Thus, analyses of the effects of different medication types on the hippocampus, precentral gyrus, and frontal pole volumes did not reveal a significant difference. Further studies are necessary to explore possible effects of medication.

Finally, the uncorrected threshold used in the present study may not fully protect against results due to chance and the results may include false positives. Therefore, the significant clusters found in the present study need to be validated further.

## Conclusions

We found that relative to the HCs, the OCD patients showed significant decreased GM volume in the right hippocampus, and increased GM volume in the left precentral gyrus. The OCD patients with the L_A_ allele carriers of 5-HTTLPR polymorphism exhibited a statistical tendency of reduction of GM volumes in the right frontal pole compared to the HCs with the L_A_ allele carriers. Our preliminary findings suggest that a variation of the 5-HTTLPR polymorphism might affect brain morphology differently in OCD patients and HCs in the right frontal pole volumes.

## Abbreviations

OCD: obsessive-compulsive disorder
CSTC: cortico–striatal–thalamo–cortical
VNTR: a variable number of tandem repeat
5-HTTLPR: serotonin transporter polymorphism
GM: gray matter
HCs: healthy controls
VBM: voxel-based morphometry
OFC: orbitofrontal cortex
DLPFC: dorsolateral prefrontal cortex
FEF: frontal eye fields
ACC: anterior cingulate cortex
PET: positron emission tomography
fMRI: functional magnetic resonance imaging
SCID: structured clinical interview for DSM-IV
Y-BOCS: Yale-Brown Obsessive Compulsive Scale
HDRS: Hamilton Depression Rating Scale
MNI: Montreal Neurological Institute
WM: white matter
PCR: polymerase chain reaction
FWE: family-wise error
IQ: intelligence quotient
